# Historical Prevalence and Distribution of Avian Influenza Virus A(H7N9) among Wild Birds

**DOI:** 10.3201/eid1912.130649

**Published:** 2013-12

**Authors:** Sarah H. Olson, Martin Gilbert, Ming Chu Cheng, Jonna A.K. Mazet, Damien O. Joly

**Affiliations:** Wildlife Conservation Society Canada, Nanaimo, British Columbia, Canada (S.H. Olson, D.O. Joly);; University of Wisconsin, Madison, Wisconsin, USA (S.H. Olson);; Wildlife Conservation Society, Bronx, New York, USA (M. Gilbert);; Animal Health Research Institute, New Taipei City, Taiwan (M.C. Cheng);; University of California, One Health Institute, Davis, California, USA (J.A.K. Mazet)

**Keywords:** expedited, population surveillance, influenza, influenza A virus, H7N9 subtype, wild animals, wild birds, avian, Asia, viruses, zoonoses, viruses

## Abstract

We examined 48 published studies for which sample sizes could be ascertained to determine the historic prevalence of influenza A(H7N9) virus in wild bird populations and reviewed GenBank data to further establish its distribution. Low prevalence (0.0093%) in Asia suggests > 30,000 samples would be required to detect the H7N9 subtype in wild birds.

Beginning in February 2013, and ongoing at publication of this article, infections with the zoonotic virus, influenza A(H7N9), have caused serious illness in humans in provinces of southeastern China. On April 4, the China Animal Disease Control Centre announced that the virus had been detected in samples collected from a pigeon and chickens at a market in Shanghai (*1*,*2*). On April 17, the virus was detected in a sample from a wild pigeon in Nanjing, Jiangsu Province (*3*). Chen et al. concluded that humans were infected by domestic birds (*1*); no human-to-human transmission was detected or suspected (*4*). The structure of the hemagglutinin (HA) protein in the virus and the lack of reports of severe disease in poultry indicate that the virus exhibits characteristics of low pathogenicity in birds (*5*,*6*). Recent phylogenetic analysis indicates that the HA segment of the H7N9 subtype is closely related to a strain that was isolated from domestic ducks in Zhejiang, China, in 2011. The neuraminidase (NA) gene of the H7N9 subtype is closely related to that of a strain that was isolated from wild bird samples in South Korea in a location adjacent to a domestic bird production facility; additionally, 6 internal genes are closely related to those of an A(H9N2) virus isolated from a brambling (*Fringilla montifringilla*) sample during 2012 in Beijing, China (*7*,*8*).

Little information exists on the status of A(H7N9) virus in wild birds to assess their potential as sources of human infection and disseminators of the virus to new areas. Here we report the historic distribution and prevalence of H7N9 subtypes among wild birds preceding this outbreak. This subtype was not known to cause disease in humans until the outbreak during February in China. We also examine the prevalence of individual H7, N9, and H9N2 subtypes in Asia. Finally, we estimate the sample size necessary to detect this low pathogenicity strain of avian influenza virus in wild birds.

## The Study

To determine prevalence of H7, N9, H7N9, and H9N2 subtypes, we reviewed 48 peer-reviewed avian influenza surveillance studies in which sample sizes were stated and subtypes were nonselectively detected by using sequence analysis, reverse transcription PCR, or hemagglutination inhibition and neuraminidase inhibition assays. Data from these studies are summarized in the Technical Appendix. These included 9 studies conducted in Asia, 12 in Europe, 4 in Africa, 3 in Australia, 17 in North America, and 3 in Latin America. Extended datasets from peer-reviewed studies in Mongolia and Taiwan were provided by M. Gilbert and M.C. Cheng, respectively. The studies sampled birds during 1976–2012.

To further establish the geographic distribution of known H7N9 subtypes, we reviewed GenBank records downloaded on April 26, 2013, for HA or NA segments isolated from birds (*9*). We included a partially sequenced HA gene (1,676 bp [GenBank accession no. JN244232]) from A/wild bird/Korea/A3/2011 in our comparison (Table) after evaluating the published phylogenetic trees (*8*).

**Table:** GenBank nucleotide sequences of H7N9 samples, country of origin, hosts, and wild or domesticated status*

HA GenBank accession no.	NA GenBank accession no.	Year	Host (family/genus/species)	Location	Status
KC899669	KC899671	2013	Chicken (*Gallus gallus*)	China	Domestic
GU060482	GU060484	2009	Goose (Anatidae)	Czech Republic	Domestic
HQ244415	HQ244417	2009	Goose (Anatidae)	Czech Republic	Domestic
CY067670	CY067672	2008	Blue-winged teal (*Anas discors*)	Guatemala	Wild
CY067678	CY067680	2008	Blue-winged teal (*Anas discors*)	Guatemala	Wild
AB813056	ND	2011	Mallard (*Anas platyrhynchos*)	Japan	Unknown
AB481212	AB481213	2008	Wild duck (Anatidae)	Mongolia	Wild
JN244232†	JN244223	2011	Wild bird	South Korea	Wild
ND	JX679164	2008	Wild duck (Anatidae)	South Korea	Wild
HQ244409	HQ244407	2008	Common teal (*Anas creccca*)	Spain	Wild
AY999981	ND	2002	Mallard (*Anas platyrhynchos*)	Sweden	Wild
CY024818	CY024820	2006	Blue-winged teal (*Anas discors*)	USA, Ohio	Wild
JX899805	ND	2011	Goose (Anatidae)	USA, Nebraska	Unknown
JX899803	ND	2011	Guinea fowl (Galliformes)	USA, Nebraska	Domestic
CY133649	CY133651	2011	Northern shoveler (*Anas clypeata*)	USA, Mississippi	Wild
EU684261	ND	2000	Ruddy turnstone (*Arenaria interpres*)	USA, Delaware	Wild
CY127253	CY127255	1995	Ruddy turnstone (*Arenaria interpres*)	USA, Delaware	Wild
CY014786	CY014788	1988	Turkey (*Meleagris* spp*.*)	USA, Minnesota	Wild/domestic‡

*HA, hemagglutinin sequence; NA, neuraminidase sequence; ND, no data were available for this variable. †Partial sequence. ‡Insufficient information was provided to determine status.

Apparent prevalence was calculated as the (no. positive samples)/(no. tested) × 100%. The regional estimate for Asia was an unweighted calculation based on the sum of all positive samples and all tested birds, irrespective of detection biases that may have arisen from different wild bird surveillance systems. We determined the minimum sample size to detect at least 1 positive sample based on a 0.05 level of significance (*10*).

## Results

Influenza H7N9 subtypes have been identified among wild birds globally (but not necessarily sequenced or submitted to GenBank) by isolation and by using reverse transcription PCR. The H7N9 subtype has been reported among wild birds from Delaware (USA)/Alberta (Canada), Guatemala, Spain, Sweden, Egypt, Mongolia, and Taiwan (Technical Appendix Table 1). In these 48 studies, subtype H7N9 has not been detected in wild birds in these locations in Asia: Russia (combined sample size 7,353), Japan (4,335), South Korea (28,214), or China (158) (Technical Appendix Table 2); furthermore, when subtype H7N9 was detected in Asia, its prevalence was low (Technical Appendix Table 2).

In countries within Asia, <0.1% of samples from wild birds tested positive for any H7 subtype; <0.05% tested positive for any N9 subtype; <0.01% tested positive for an H7N9 strain, and <0.02% tested positive for an H9N2 strain (Technical Appendix Table 2). Assuming an apparent prevalence of 0.01%, we estimate that >30,000 birds would have to be sampled to detect 1 bird that was H7N9-positive with a 95% probability. To similarly detect 1 bird that was positive for H7, N9, or the H9N2 subtype in Asia, >4,000, 7,000, or 19,000 samples from birds, respectively, would be required.

Since 1988, the HA- and NA-producing genes of avian influenza subtype H7N9 have been deposited in GenBank 12 times, mainly representing isolates collected from wild bird hosts (Table). In Asia, before this outbreak, an H7N9 strain was sequenced from a wild bird in South Korea that was sampled during 2011 in a migratory bird habitat adjacent to duck farms (*7*) and also during 2011 in a sample from a mallard duck of unknown status from Japan. In 2008, the other H7N9 strain sequences collected in Asia were from a wild duck that was sampled in South Korea and from a wild bird sampled in Mongolia. All virus sequences were obtained from ducks and domestic geese, with the exception of a chicken in China and the following from birds in the United States: a turkey in Minnesota, a guinea fowl in Nebraska, and ruddy turnstones (*Arenaria interpres*) sampled in Delaware during 1995 and 2000. Eight of the complete HA and NA genetic sequences are attributed to wild birds, 3 are attributed to domestic birds, and 1 is attributed to a bird that could not be identified as wild or domestic because insufficient information was available.

## Conclusions

Variation in the methods used in each study makes a precise calculation of H7N9 subtype prevalence in all wild birds impossible to determine, but given the available data, we conclude that the occurrence of the H7N9 subtype in wild bird populations is rare. We also conclude that sample sizes adequate to detect the virus among wild birds will be in the tens of thousands. Publishing the sample size and genus and species of wild birds tested in China will provide a better estimate of the prevalence among these birds related to this outbreak, especially because wild song birds have been hypothesized to be a possible reservoir (*11*). Wild birds are recorded as the predominant source of H7N9 sequences, but this may be an outcome of sampling bias. Because virologists typically focus on highly pathogenic strains in humans and domestic birds, and an H7N9 subtype was not recognized as highly pathogenic, the H7N9 strains were not was not tested for as frequently in wild birds. The HA/NA subtype concept we used for this analysis is archaic, omitting the contributions of internal protein genes to the biology of a virus; unfortunately, it is the only widespread typing system available for influenza viruses. Subsequently, the best historic prevalence estimate of the circulating internal genes is based on the H9N2 subtype.

Infection with the H7N9 subtype may prove challenging to control by culling birds, because infected domestic flocks may be asymptomatic. In wild bird populations, low pathogenicity strains are likely to be sustained longer than highly pathogenic strains, which have been unable to persist in wild populations in the absence of introductions from a domestic reservoir (*12*). Further research should focus on identifying sequences within the new H7N9 genome that are linked to increased human pathogenicity and transmissibility and on conducting surveillance to detect these markers in viruses carried by both domestic and wild birds (*13*).

In summary, we present evidence that wild bird surveillance for the novel influenza A(H7N9) virus will require large sample sizes. Given the low likelihood of detection, risk-based surveillance is recommended. Ruling out wild birds as a continuing source of infection for domestic birds or humans will be critical to informing strategies to control the spread of this emerging zoonotic disease.

Technical Appendix Historic global distribution and prevalence of influenza A (H7N9) in wild birds and prevalence of subtypes H7, N9, H7N9, and H9N2 in Asia, supported by a review of 48 studies.
