# Supplementary material for: Historical Prevalence and Distribution of Avian Influenza Virus A(H7N9) among Wild Birds
Source: Emerg Infect Dis. 2013 Dec;19(12):2031–3. doi: 10.3201/eid1912.130649 (PMC3840878; doi:10.3201/eid1912.130649)
Supplement: Technical Appendix — Historic global distribution and prevalence of influenza A (H7N9) in wild birds and prevalence of subtypes H7, N9, H7N9, and H9N2 in Asia, supported by a review of 48 studies. [file 13-0649-Techapp-s1.pdf]

Article DOI: http://dx.doi.org/10.3201/eid1912.132013

Historic Prevalence and Distribution of Avian Influenza Virus A(H7N9) among Wild Birds

Technical Appendix

Technical Appendix Table 1. Global distribution and prevalence of influenza H7N9 virus in wild birds

Country/state (*reference*)	Years sampled	No. tested	No. (%) H7N9	H7N9 virus-positive birds	Population sampled*: no. positive for any avian influenza virus/no. captured (%)
Guatemala (*1*)	2006–2010	256	1 (0.39)	Blue-winged teal (*Anas discors*)	Anatidae: 28/234 (12); Picidae: 1/21 (4.8); Tyrannidae: 1/1 (100)
Spain (*2*)	2005–2007	1,435	2 (0.14)	Mallards (*Anas platyrhynchos*)	Anseriformes: 29/628 (4.6); Ciconiformes: 3/308 (1); Charadriiformes: 0/217; Gruiformes: 3/180 (1.7); Columbiformes: 0/31; Pelecaniformes: 0/29; Passeriformes: 0/24; Phoenicopteriformes: 2/7 (28.6); Other: 0/11
Spain (*3*)	2005–2009	1,236	1 (0.081)	Common teal (*Anas crecca*)	Anatidae: 54/686 (7.9%); Laridae 2/256 (0.8%); Phoenicopteridae: 4/154 (2.5%); Rallidae: 1/80 (1.3%); Ardeidae: 0/12; Porphyridae: 0/12; Porphyridae: 0/12; Columbidae: 0/9; Procellariidae: 0/9; Phalacrocoracidae: 0/6; Scolopacidae: 0/4; Recurvirostridae: 0/2; Sternidae: 0/2; Alcedinidae: 0/1; Fringillidae: 0/1; Phasianidae: 0/1; Stercorariidae: 0/1.
Egypt (*4*)	2003–2007	6,070	2 (0.033)	Northern shovelers (*Anas clypeata*)	Anatidae majority: not reported.
Delaware, USA (*5*)	2000–2005	6,340	2 (0.032)	Mix of shorebirds and ducks	*Arenaria interpres*: 262/2368 (11%); *Calidris canutus*: 15/1993 (0.75); *Calidris alba*: 8/745 (1.1); *Calidris alpine*: 1/377 (0.26); *Calidris pusilla*: 4/439 (0.91%); *Limnodromus griseus*:1/157 (0.64%); *Calidris minutilla*: 0/127; *Larus atricilla*: 1/78 (1.3%); *Rynchops niger*: 0/49; *Larus argentatus*: 0/5; *Tringa semipalmatus*: 0/2
Mongolia (*6*)	2005–2009	3,950	1 (0.025)	Wild duck, goose, swan or gull	Not reported: *Cygnus cygnus*; *Anser indicus*; *Bucephala clangula*; *Tadorna ferruginea*; *Anas platyrhynchos*; *Anas strepera*; Laridae
Taiwan (*7*) + this study	1998–2011	44,786	8 (0.018)	Wild ducks	Anatidae 229/20812 (1.1%); Shorebirds 3/6435 (0.05%); Laridae (2/617, 0.32%); Ardeidae (2/825, 0.24%); other birds (1/598, 0.17%)
Delaware, USA, & Alberta, Canada (*8*)	1976–2001	17,732	2 (0.011)	Mix of shorebirds and ducks	Anatidae (2989/13466, 22.2%); Charadriiformes—*Larus atricilla* and *L. argentatus* majority (606/4266, 14.2%)

*Populations sampled represent individual published studies in correlating countries/states.

Technical Appendix Table 2. Influenza H7, N9, H7N9, and H9N2 prevalence in Asia*

Country (*reference*)	Years monitored	No. tested	No. positive (%)				Population sampled:** no. positive for any avian influenza virus/no. captured (%)
			H7	N9	H7N9	H9N2	
Mongolia (*6*)	2005–2009	3,950	2 (0.0506)	1 (0.0253)	1 (0.0253)	0	Not reported: *Anser indicus*; *Anas platyrhynchos*; *Anas strepera*; *Bucephala clangula*; *Cygnus cygnus*; *Tadorna ferruginea*; Laridae
Taiwan (*7*) + this study	1998–2011	44,786	49 (0.109)	23 (0.0514)	8 (0.0179)	0	Anatidae: 229/20812 (1.1); shorebirds: 3/6435 (0.05%); Laridae: 2/617 (0.32%); Ardeidae: 2/825 (0.24%); other birds: 1/598 (0.17%)
Asia (Kazakhstan, Mongolia, & Russia) (*9*)	2003–2009	2,604	0	0	0	0	Anseriformes: 9/604 (1.5%); Charadriiformes 1/684 (0.14%); Passeriformes: 2/674 (0.30); Ciconiiformes: 0/300; Gruiformes: 0/118; Podicipediformes 1/45 (2.2%); Pelecaniformes: 3/95 (3.2%); Falconiformes: 1/42 (2.4%); Galliformes: 0/16; Columbiformes: 0/18; Coraciiformes: 0/3; Cuculiformes: 0/2; Piciformes: 0/2; Strigiformes: 0/1
Russia (*10*)	2008	5,678	0	0	0	0	Anatidae: 30/2017 (1.5%); Araeidae: 1/81 (1.2%); Laridae: 10/1,553 (0.64%); Rallidae: 1/164 (0.61); Podicipediformes: 0/64; Scolopacidae: 0/334; Alcidae: 0/19; Charadriidae: 0/190; Recurvirostridae: 0/1; Stercorariidae: 0/1; Columbiformes: 0/25; Passeriformes: 0/989; Galliformes:(0/3; Strigiformes: 0/5; Accipitriformes: 0/8; Coraciiformes: 0/26; Apodiformes: 0/1; Piciformes: 0/3; Guidae: 0/1; Gaviiformes: 0/1; Pelecaniformes: 0/187; Procellariiformes: 0/5
Japan (*11)*	2001–2008	4,335	0	6 (0.138)	0	1 (0.0231)	Anatidae: 41/4309 (0.95%); Laridae 0/26
South Korea (*12*)	2003–2008	28,214	13 (0.0461)	7 (0.0248)	0	6 (0.0213)	Anseriformes: 225/28214 (0.8%)
China (*13*)	2005–2006	158	0	0	0	0	Anatidae: 20/144 (15%); Ardeidae: 0/2; Accipitridae: 0/4; Charadriidae: 0/1; Corvidae: 0/1; Falconidae: 0/2; Gruidae: 0/2; Rallidae: 0/1; Phasianidae: 0/1; Upupidae: 0/1
Iran (*14*)	2003–2007	1,146	2 (0.175)	2 (0.175)	0	9 (0.785)	Podicipedidae: 0/19; Phalacrocoracidae: 0/14; Ardeidae: 0/22; Phoenicopteridae: 0/12; Anatidae: 31/745 (4.2%); Rallidae: 4/234, (1.8%); Recurvirostridae: 0/6; Charadriidae: 0/17; Scolopacidae: 0/51; Laridae: 0/25; Sternidae: 0/1
Mongolia (*15*) + this study	2009–2012	5,831	6 (0.103)	2 (0.0343)	0	0	Anatidae: (80/5731 (1.4%); Laridae: 0/100
Asia total	1998–2012	96,702	72 (0.0745)	41 (0.0424)	9 (0.0093 1)	16 (0.0166)	NA†

*Populations sampled represent individual published studies in correlating countries/states.
†NA, not applicable.

References

1. González-Reiche AS, Morales-Betoulle ME, Alvarez D, Betoulle JL, Müller ML, Sosa SM, et al. Influenza A viruses from wild birds in Guatemala belong to the North American lineage. PLoS ONE. 2012;7:e32873. PubMed http://dx.doi.org/10.1371/journal.pone.0032873

2. Pérez-Ramírez E, Gerrikagoitia X, Barral M, Höfle U. Detection of low pathogenic avian influenza viruses in wild birds in Castilla-La Mancha (south central Spain). Vet Microbiol. 2010;146:200–8. PubMed http://dx.doi.org/10.1016/j.vetmic.2010.05.008

3. Busquets N, Alba A, Napp S, Sánchez A, Serrano E, Rivas R, et al. Influenza A virus subtypes in wild birds in North-Eastern Spain (Catalonia). Virus Res. 2010;149:10–8. PubMed http://dx.doi.org/10.1016/j.virusres.2009.12.005

4. Soliman A, Saad M, Elassal E, Amir E, Plathonoff C, Bahgat V, et al. Surveillance of avian influenza viruses in migratory birds in Egypt, 2003-09. J Wildl Dis. 2012;48:669–75. PubMed

5. Hanson BA, Luttrell MP, Goekjian VH, Niles L, Swayne DE, Senne DA, et al. Is the occurrence of avian influenza virus in Charadriiformes species and location dependent? J Wildl Dis. 2008;44:351–61. PubMed

6. Tseren-Ochir E-O, Damdinjav B, Sharkhuu T, Kang HM, Sakoda Y, Purevsuren B, et al. Epidemiology of avian influenza viruses in wild birds in Mongolia. Int J Infect Dis. 2010;14:e164 http://dx.doi.org/10.1016/j.ijid.2010.02.1846.

7. Cheng MC, Lee MS, Ho YH, Chyi WL, Wang CH. Avian influenza monitoring in migrating birds in Taiwan during 1998–2007. Avian Dis. 2010;54:109–14. PubMed http://dx.doi.org/10.1637/8960-061709-Reg.1

8. Krauss S, Walker D, Pryor SP, Niles L, Chenghong L, Hinshaw VS, et al. Influenza A viruses of migrating wild aquatic birds in North America. Vector Borne Zoonotic Dis. 2004;4:177–89. PubMed http://dx.doi.org/10.1089/vbz.2004.4.177

9. Marchenko VY, Alekseev AY, Sharshov KA, Petrov VN, Silko NY, Susloparov IM, et al. Ecology of influenza virus in wild bird populations in Central Asia. Avian Dis. 2012;56:234–7. PubMed http://dx.doi.org/10.1637/9834-061611-ResNote.1

10. Sivay MV, Sayfutdinova SG, Sharshov KA, Alekseev AY, Yurlov AK, Runstadler J, et al. Surveillance of influenza A virus in wild birds in the Asian portion of Russia in 2008. Avian Dis. 2012;56:456–63. PubMed http://dx.doi.org/10.1637/9868-080111-Reg.1

11. Fujimoto Y, Ito H, Shivakoti S, Nakamori J, Tsunekuni R, Otsuki K, et al. Avian influenza virus and paramyxovirus isolation from migratory waterfowl and shorebirds in San-in district of western Japan from 2001 to 2008. J Vet Med Sci. 2010;72:963–7. PubMed http://dx.doi.org/10.1292/jvms.10-0012

12. Kang HM, Jeong OM, Kim MC, Kwon JS, Paek MR, Choi JG, et al. Surveillance of avian influenza virus in wild bird fecal samples from South Korea, 2003–2008. J Wildl Dis. 2010;46:878–88. PubMed

13. Zeng X, Hua Y, Li X, Zhang Z. Monitoring influenza A virus and Newcastle disease virus in migratory waterfowls in Sanjiang natural reserve of Heilongjiang Province. Wei Sheng Wu Xue Bao. 2008;48:1403–7. PubMed

14. Fereidouni SR, Werner O, Starick E, Beer M, Harder TC, Aghakhan M, et al. Avian influenza virus monitoring in wintering waterbirds in Iran, 2003–2007. Virol J. 2010;7:43. PubMed http://dx.doi.org/10.1186/1743-422X-7-43

15. Gilbert M, Jambal L, Karesh WB, Fine A, Shiilegdamba E, Dulam P, et al. Highly pathogenic avian influenza virus among wild birds in Mongolia. PLoS ONE. 2012;7:e44097. PubMed http://dx.doi.org/10.1371/journal.pone.0044097

16. Pereda AJ, Uhart M, Perez AA, Zaccagnini ME, La Sala L, Decarre J, et al. Avian influenza virus isolated in wild waterfowl in Argentina: evidence of a potentially unique phylogenetic lineage in South America. Virology. 2008;378:363–70. PubMed http://dx.doi.org/10.1016/j.virol.2008.06.010

17. Escudero G, Munster VJ, Bertellotti M, Edelaar P. Perpetuation of avian influenza in the Americas: examining the role of shorebirds in Patagonia. The Auk. 2008;125:494–5 http://dx.doi.org/10.1525/auk.2008.2408.2.

18. Haynes L, Arzey E, Bell C, Buchanan N, Burgess G, Cronan V, et al. Australian surveillance for avian influenza viruses in wild birds between July 2005 and June 2007. Aust Vet J. 2009;87:266–72. PubMed http://dx.doi.org/10.1111/j.1751-0813.2009.00446.x

19. Peroulis I, O'Riley K. Detection of avian paramyxoviruses and influenza viruses amongst wild bird populations in Victoria. Aust Vet J. 2004;82:79–82. PubMed http://dx.doi.org/10.1111/j.1751-0813.2004.tb14650.x

20. Mackenzie JS, Edwards EC, Holmes RM, Hinshaw VS. Isolation of ortho- and paramyxoviruses from wild birds in Western Australia, and the characterization of novel influenza A viruses. Aust J Exp Biol Med Sci. 1984;62:89–99. PubMed http://dx.doi.org/10.1038/icb.1984.9

21. Munster VJ, Baas C, Lexmond P, Waldenström J, Wallensten A, Fransson T, et al. Spatial, temporal, and species variation in prevalence of influenza A viruses in wild migratory birds. PLoS Pathog. 2007;3:e61. PubMed http://dx.doi.org/10.1371/journal.ppat.0030061

22. Gaidet N, Dodman T, Caron A, Balanca G, Desvaux S, Goutard F, et al. (2007) Influenza surveillance in wild birds in Eastern Europe, the Middle East, and Africa: preliminary results from an ongoing FAO-led survey. J Wildl Dis.. 2007;43: Suppl : S22–8. http://www.jwildlifedis.org/content/43/3_Supplement/S22.full

23. Lebarbenchon C, Chang CM, van der Werf S, Aubin JT, Kayser Y, Ballesteros M, et al. Influenza A virus in birds during spring migration in the Camargue, France. J Wildl Dis. 2007;43:789–93. PubMed

24. Hlinak A, Mühle RU, Werner O, Globig A, Starick E, Schirrmeier H, et al. A virological survey in migrating waders and other waterfowl in one of the most important resting sites of Germany. J Vet Med B Infect Dis Vet Public Health. 2006;53:105–10. PubMed http://dx.doi.org/10.1111/j.1439-0450.2006.00935.x

25. Süss J, Schäfer J, Sinnecker H, Webster RG. Influenza virus subtypes in aquatic birds of eastern Germany. Arch Virol. 1994;135:101–14. PubMed http://dx.doi.org/10.1007/BF01309768

26. De Marco MA, Foni E, Campitelli L, Delogu M, Raffini E, Chiapponi C, et al. Influenza virus circulation in wild aquatic birds in Italy during H5N2 and H7N1 poultry epidemic periods (1998 to 2000). Avian Pathol. 2005;34:480–5. PubMed http://dx.doi.org/10.1080/03079450500368185

27. Terregino C, De Nardi R, Guberti V, Scremin M, Raffini E, Martin AM, et al. Active surveillance for avian influenza viruses in wild birds and backyard flocks in Northern Italy during 2004 to 2006. Avian Pathol. 2007;36:337–44. PubMed http://dx.doi.org/10.1080/03079450701488345

28. Henriques AM, Fagulha T, Barros SC, Ramos F, Duarte M, Luís T, et al. Multiyear surveillance of influenza A virus in wild birds in Portugal. Avian Pathol. 2011;40:597–602. PubMed http://dx.doi.org/10.1080/03079457.2011.618943

29. Tolf C, Bengtsson D, Rodrigues D, Latorre-Margalef N, Wille M, Figueiredo ME, et al. Birds and viruses at a crossroad—surveillance of influenza A virus in Portuguese waterfowl. PLoS ONE. 2012;7:e49002. PubMed http://dx.doi.org/10.1371/journal.pone.0049002

30. Cumming GS, Caron A, Abolnik C, Cattoli G, Bruinzeel LW, Burger CE, et al. The ecology of influenza A viruses in wild birds in southern Africa. EcoHealth. 2011;8:4–13. PubMed http://dx.doi.org/10.1007/s10393-011-0684-z

31. Baumer A, Feldmann J, Renzullo S, Müller M, Thür B, Hofmann MA. Epidemiology of avian influenza virus in wild birds in Switzerland between 2006 and 2009. Avian Dis. 2010;54:875–84. PubMed http://dx.doi.org/10.1637/9119-110209-Reg.1

32. Kulak MV, Ilinykh FA, Zaykovskaya AV, Epanchinzeva AV, Evstaphiev IL, Tovtunec NN, et al. Surveillance and identification of influenza A viruses in wild aquatic birds in the Crimea, Ukraine (2006–2008). Avian Dis. 2010;54:1086–90. PubMed http://dx.doi.org/10.1637/9272-020510-ResNote.1

33. Simulundu E, Ishii A, Igarashi M, Mweene AS, Suzuki Y, Hang'ombe BM, et al. Characterization of influenza A viruses isolated from wild waterfowl in Zambia. J Gen Virol. 2011;92:1416–27. PubMed

34. Hinshaw VS, Wood JM, Webster RG, Deibel R, Turner B. Circulation of influenza viruses and paramyxoviruses in waterfowl originating from two different areas of North America. Bull World Health Organ. 1985;63:711–9. PubMed

35. Krauss S, Pryor SP, Raven G, Danner A, Kayali G, Webby RJ, et al. Respiratory tract versus cloacal sampling of migratory ducks for influenza A viruses: are both ends relevant? Influenza Other Respi Viruses. 2013;7:93–6. PubMed http://dx.doi.org/10.1111/j.1750-2659.2012.00359.x

36. Wille M, Robertson GJ, Whitney H, Ojkic D, Lang AS. Reassortment of American and Eurasian genes in an influenza A virus isolated from a great black-backed gull (Larus marinus), a species demonstrated to move between these regions. Arch Virol. 2011;156:107–15. PubMed http://dx.doi.org/10.1007/s00705-010-0839-1

37. Ito T, Okazaki K, Kawaoka Y, Takada A, Webster RG, Kida H. Perpetuation of influenza A viruses in Alaskan waterfowl reservoirs. Arch Virol. 1995;140:1163–72. PubMed http://dx.doi.org/10.1007/BF01322743

38. Ramey AM, Pearce JM, Flint PL, Ip HS, Derksen DV, Franson JC, et al. Intercontinental reassortment and genomic variation of low pathogenic avian influenza viruses isolated from northern pintails (*Anas acuta*) in Alaska: examining the evidence through space and time. Virology. 2010;401:179–89. PubMed http://dx.doi.org/10.1016/j.virol.2010.02.006

39. Koehler AV, Pearce JM, Flint PL, Franson JC, Ip HS. Genetic evidence of intercontinental movement of avian influenza in a migratory bird: the northern pintail (*Anas acuta*). Mol Ecol. 2008;17:4754–62. PubMed http://dx.doi.org/10.1111/j.1365-294X.2008.03953.x

40. Ramey AM, Pearce JM, Reeves AB, Franson JC, Petersen MR, Ip HS. Evidence for limited exchange of avian influenza viruses between seaducks and dabbling ducks at Alaska Peninsula coastal lagoons. Arch Virol. 2011;156:1813–21. PubMed http://dx.doi.org/10.1007/s00705-011-1059-z

41. Siembieda JL, Johnson CK, Cardona C, Anchell N, Dao N, Reisen W, et al. Influenza A viruses in wild birds of the Pacific flyway, 2005–2008. Vector Borne Zoonotic Dis. 2010;10:793–800. PubMed http://dx.doi.org/10.1089/vbz.2009.0095

42. Stallknecht DE, Shane SM, Zwank PJ, Senne DA, Kearney MT. Avian influenza viruses from migratory and resident ducks of coastal Louisiana. Avian Dis. 1990;34:398–405. PubMed http://dx.doi.org/10.2307/1591427

43. Wilcox BR, Knutsen GA, Berdeen J, Goekjian V, Poulson R, Goyal S, et al. Influenza-A viruses in ducks in northwestern Minnesota: fine scale spatial and temporal variation in prevalence and subtype diversity. PLoS ONE. 2011;6:e24010. PubMed http://dx.doi.org/10.1371/journal.pone.0024010

44. Lebarbenchon C, Sreevatsan S, Ramakrishnan MA, Poulson R, Goekjian V, Di Matteo JJ, et al. Influenza A viruses in American White Pelican (*Pelecanus erythrorhynchos*). J Wildl Dis. 2010;46:1284–9. PubMed

45. Krauss S, Walker D, Pryor SP, Niles L, Chenghong L, Hinshaw VS, et al. Influenza A viruses of migrating wild aquatic birds in North America. Vector Borne Zoonotic Dis. 2004;4:177–89. PubMed http://dx.doi.org/10.1089/vbz.2004.4.177

46. Slemons RD, Shieldcastle MC, Heyman LD, Bednarik KE, Senne DA. Type A influenza viruses in waterfowl in Ohio and implications for domestic turkeys. Avian Dis. 1991;35:165–73. PubMed http://dx.doi.org/10.2307/1591309

47. Alfonso CP, Cowen BS, van Campen H. Influenza A viruses isolated from waterfowl in two wildlife management areas of Pennsylvania. J Wildl Dis. 1995;31:179–85. PubMed

48. Hanson BA, Swayne DE, Senne DA, Lobpries DS, Hurst J, Stallknecht DE. Avian influenza viruses and paramyxoviruses in wintering and resident ducks in Texas. J Wildl Dis. 2005;41:624–8. PubMed http://www.jwildlifedis.org/content/41/3/624.long
